# Supplementary material for: The N-Terminus Makes the Difference: Impact of Genotype-Specific Disparities in the N-Terminal Part of The Hepatitis B Virus Large Surface Protein on Morphogenesis of Viral and Subviral Particles
Source: Cells. 2020 Aug 13;9(8):1898. doi: 10.3390/cells9081898 (PMC7463600; doi:10.3390/cells9081898)
Supplement: Supplementary file 1 [file cells-09-01898-s001.pdf]

Table S1: Primers used for mutagenesis PCR.

Name	Primer Sequences	Remarks
Δ11aa_fw	CCATATTCTTGGGAACAAGAGCTACAGCTAGGGAGGTTGG	The first ATG of PreS1 was mutated to TAG.
Δ11aa_rev	GCGAGGTTTTGATGACCAACCTCCCTAGCTGTAGC	
(+)11aa(GtA)_fw	TCACCATATTCTTGGGAACAAGATCTACAGCATGGGAGGTTGGTCATCAAAACCTCGC	
(+)11aa(GtA)_rev	GCTGGTGGAAAGATTCTGCCCCATGCCTTTGCGAGGTTTTGATGACCAAC	
(+)10aa(GtE)_fw	ACCATATTCTTGGGAACAAGATCTACAGCATGGGGCTTTCTTGGACGGTCCCTCTCG	
(+)10aa(GtE)_rev	TTGCTGGTGGAAAGATTCTGCCCCCATTCGAGAGGGACCGTCCAAG	
Δaa2-7_fw	GGTCACCATATTCTTGGGAACAAGAGCTACAGCATGCCTCGCAAAG	
Δaa2-7_rev	GAACAGAAAGATTCGTCCCCATGCCTTTGCGAGGCATGCTGTA	
Δaa3-8_fw	GGTCACCATATTCTTGGGAACAAGAGCTACAGCATGGGACGCAAAG	
Δaa3-8_rev	GAACAGAAAGATTCGTCCCCATGCCTTTGCGTCCCATGCTGTA	
Δaa4-9_fw	GGTCACCATATTCTTGGGAACAAGAGCTACAGCATGGGAGGAAAAGG	
Δaa4-9_rev	GAACAGAAAGATTCGTCCCCATGCCTTTTCCTCCCATGCTGTAG	
Δaa5-10_fw	GGTCACCATATTCTTGGGAACAAGAGCTACAGCATGGGAGGTTGGG	
Δaa5-10_rev	GAACAGAAAGATTCGTCCCCATGCCCCAACCTCCCATGCTGTA	
Δaa6-11_fw	GGTCACCATATTCTTGGGAACAAGAGCTACAGCATGGGAGGTTGGT	
Δaa6-11_rev	GGGAACAGAAAGATTCGTCCCCATTGACCAACCTCCCATGCTGTA	
GtA_aa3-8(GtE)_fw	GGGAACAAGAGCTACAGCATGGGACTTTCTTGGACGGTCCCTC	
GtA_aa3-8(GtE)_rev	GATTCGTCCCCATGCCTTTGCGAGGGACCGTCCAAGAAAG	
GtA_aa7-12(GtE)_fw	ACAGCATGGGAGGTTGGTCATCAACGGTCCCTCTCGAGTGGG	
GtA_aa7-12(GtE)_rev	GGTTGGGAACAGAAAGATTCGTCCCCCACTCGAGAGGGACCGT	
Δaa2-5_fw	TTTGCGGGTCACCATATTCTTGGGAACAAGAGCTACAGCATGTCAAAACCTCG	
Δaa2-5_rev	TGGGAACAGAAAGATTCGTCCCCATGCCTTTGCGAGGTTTTGACATGCTGTAG	
Δaa6-8_fw	GTCACCATATTCTTGGGAACAAGAGCTACAGCATGGGAGGTTGGTCACGCAAAGGC	
Δaa6-8_rev	CCCAGAGGGTTGGGAACAGAAAGATTCGTCCCCATGCCTTTGCGTGACCAAC	
Δaa9-11_fw	GGGAACAAGAGCTACAGCATGGGAGGTTGGTCATCAAAACCTATGGGGACGAA	
Δaa9-11_rev	GAAAGAATCCCAGAGGGTTGGGAACAGAAAGATTCGTCCCCATAGGTTTTGAT	

K7M_fw	CGGGTCACCATATTCTTGGGAACAAGAGCTACAGCACGGGAGGTTGGTCATCAAT	The first ATG of PreS1 was mutated to ACG.The lysine at position 7 was mutated to methionine.
K7M_rev	ACAGAAAGATTCGTCCCCATGCCTTTGCGAGGCATTGATGACCAACCTCCCG	
GtA_aa2-6(GtE)_fw	GGGAACAAGAGCTACAGCATGGGACTTTCTTGGAAACCTC	
GtA_aa2-6(GtE)_rev	TCCCCATGCCTTTGCGAGGTTTCCAAGAAAGTCCCAT	
GtA_aa2-12(GtE)_fw	GGGAACAAGAGCTACAGCATGGGGCTTTCTTGGACGGTCCCTCTCG	
GtA_aa2-12(GtE)_rev	GTTGGGAACAGAAAGATTCGTCCCCCATTCGAGAGGGACCGTCCAAG	
W5M_fw	ACCTTATTCTTGGGAACAAGAGCTACATCACGGGGCTTTCTATGAC	The first ATG of PreS1 was mutated to ACG.The tryptophan at position 5 was mutated to methionine.
W5M_rev	TCCCCCATTCGAGAGGGACCGTCATAGAAAGCCCCGTG	
P8M_fw	CACCTTATTCTTGGGAACAAGAGCTACATCACGGGGCTTTCTTGGACGGTCA	The first ATG of PreS1 was mutated to ACG.The proline at position 8 was mutated to methionine.
P8M_rev	GGTGGAATGATTCTTCCCCCATTCGAGCATGACCGTCCAAGAAAGCC	
W11M_fw	CACCTTATTCTTGGGAACAAGAGCTACATCACGGGGCTTTCTTGGACGGTCCCTCT	The first ATG of PreS1 was mutated to ACG.The trypophan at position 11 was mutated to methionine.
W11M_rev	AGGATTGGTGGTGGAATGATTCTTCCCCATTTCGAGAGGGACCGTCCAAGAA	

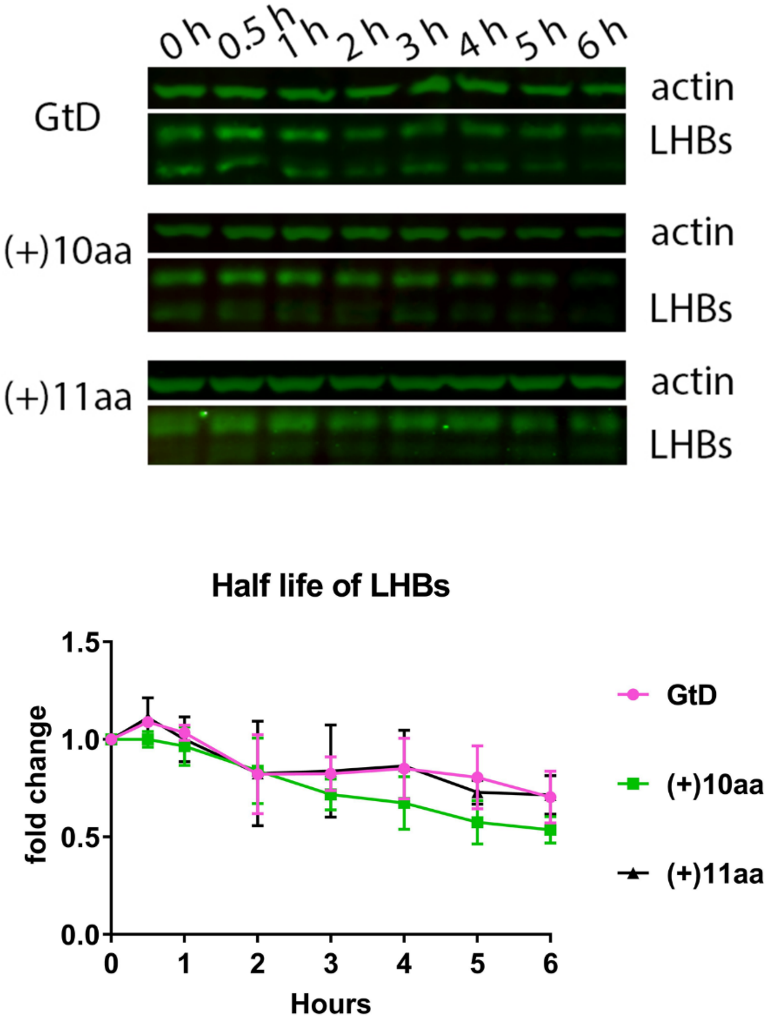

	GtD	(+)10aa	(+)11aa
Half Life	11.1	6.178	10.39

Figure S1. 11 aa-associated higher amount of LHBs does not correlate with longer protein half life. Huh7.5 cells transfected with genomes GtD and the derivatives were treated with 100 μg/ml cycloheximide (CHX) up to 6 hours. The lysates were analyzed by Western blot using a PreS1-specific serum (MA18/7). β-actin was used as loading control. The signals of LHBs and β-actin in the Western blot were quantified by Image studio lite from LI-COR Biosciences. The signal from untreated sample was standardized as 1. These quantitative data are mean values from three independent experiments.

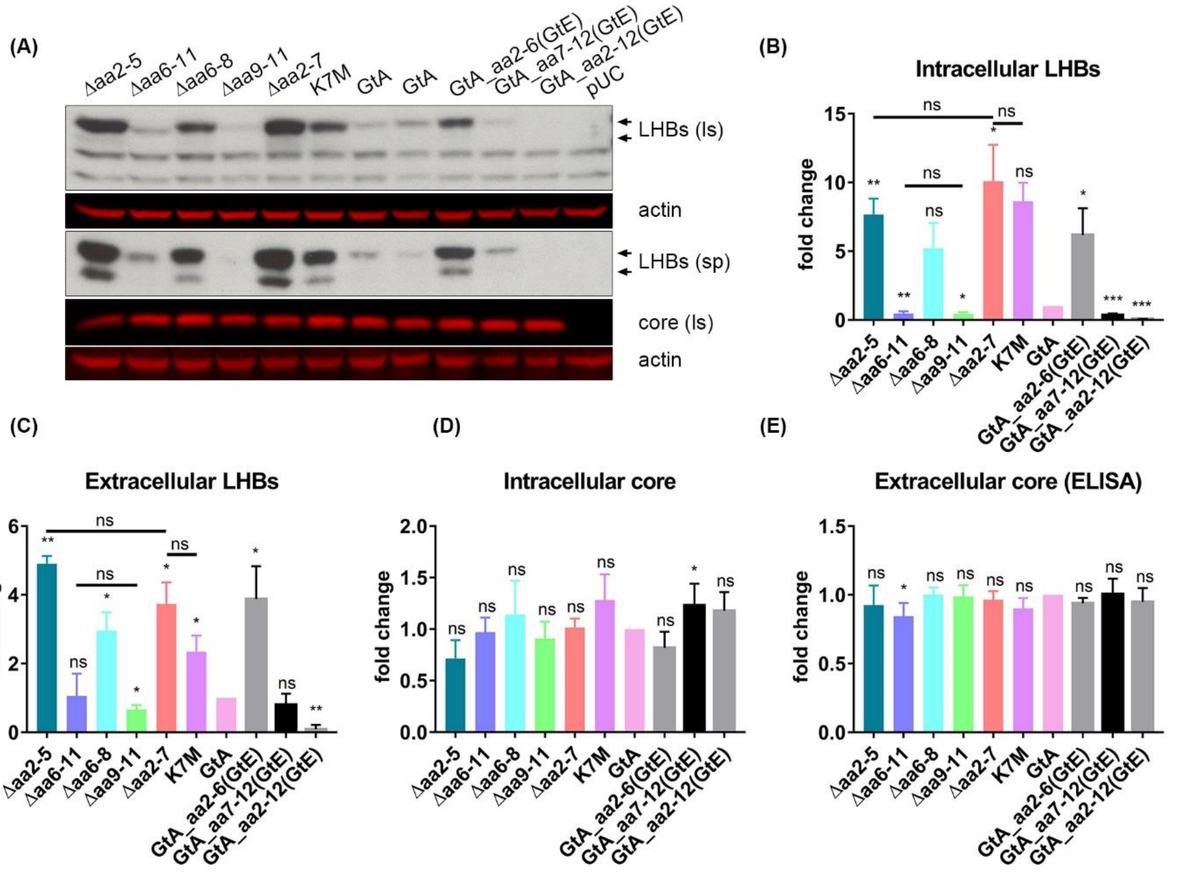

Figure S2. Deletion of only 3 aa in the GtA-derived 11 aa alters dramatically the amount of LHBs. (A) Supernatants and lysates from Huh7.5 cells transfected with genomes GtA and the derivatives were analyzed by Western blot using the PreS1-specific serum (MA18/7) and the core-specific serum (K46). β-actin was used as loading control. (B-D) The signals of LHBs and core in the Western blot were quantified by Image studio lite from LI-COR Biosciences. (E) Supernatants from Huh7.5 cells transfected with genomes GtA and the derivatives were analyzed by core ELISA. The signal from GtA was standardized as 1. Ls, lysate; sp, supernatant. These quantitative data are mean values from three independent experiments.

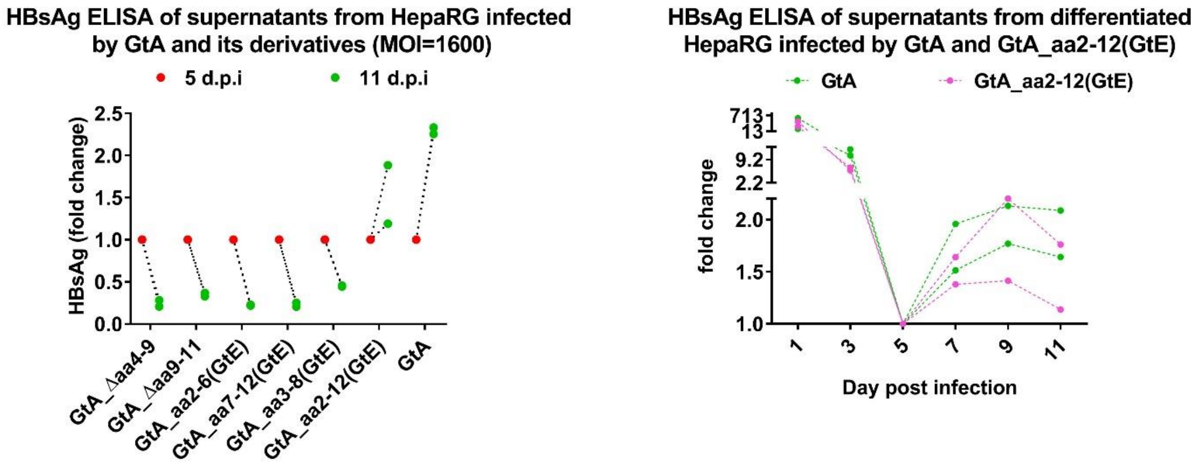

Figure S3. Substitution the N-terminal 11 aa in PreS1 domain of GtA with N-terminal 10 aa in the PreS1 domain of GtE does not impair the viral infectivity. Differentiated HepaRG were infected with HBV from GtA and its derivatives at MOI 1600. Culture supernatants collected at every two days post infection and samples from 5 d.p.i and 11 d.p.i (days post infection) were analyzed by HBsAg ELISA (left panel). To further confirm the successful infection established by virus from GtA_aa2-12(GtE), culture supernatants collected at indicated time points were analyzed by HBsAg ELISA. These data are from two independent experiments.
